# Supplementary material for: Combination of Symptom Profile, Endoscopic Findings, and Esophageal Mucosal Histopathology Helps to Differentiate Achalasia from Refractory Gastroesophageal Reflux Disease
Source: Diagnostics (Basel). 2021 Dec 13;11(12):2347. doi: 10.3390/diagnostics11122347 (PMC8700273; doi:10.3390/diagnostics11122347)
Supplement: Supplementary file 1 [file diagnostics-11-02347-s001.zip › diagnostics-1496596-supplementary.pdf]

Table S1. Clinical characteristics and histopathology of type I and type II achalasia.

	Type I	Type II	*p* value
Number of patients	24	28	
Age (years)	50.6 ± 12.2	53.7 ± 16.1	0.454
Male gender (%)	9 (37.5%)	9 (32.1%)	0.686
BMI (kg/m^2)	21.3 ± 2.9	21.6 ± 4.2	0.764
Waist (cm)	77.4 ± 9.9	75.6 ± 14.4	0.613
Symptom duration (months)	93.6 ± 93.2	44.7 ± 57.1	0.039
**Symptom profile**			
RDQ score	15.9 ± 13.2	15.4 ± 12.8	0.877
Heartburn domain	4.6 ± 4.9	4.7 ± 5.1	0.950
Dyspepsia domain	4.5 ± 5.5	4.3 ± 5.3	0.885
Regurgitation domain	6.8 ± 6.5	6.3 ± 6.6	0.429
Eckardt score	5.3 ± 2.0	5.1 ± 2.6	0.828
Dysphagia	2.5 ± 0.8	1.9 ± 1.0	0.047
Retrosternal pain	0.6 ± 0.7	0.6 ± 0.8	0.934
Regurgitation	1.3 ± 0.9	1.4 ± 1.2	0.752
Body weight loss	0.8 ± 1.0	1.1 ± 1.1	0.357
**Endoscopic findings**			
Erosive esophagitis (%)	2 (8.3%)	2 (7.1%)	0.872
Esophageal food retention (%)	20 (83.3%)	10 (35.7%)	0.001
**HRIM parameters**			
LES resting pressure (mmHg)	26.8 ± 17.9	34.4 ± 15.0	0.132
LES IRP 4s (mmHg)	18.6 ± 13.1	26.2 ± 11.1	0.041
**Histopathology**			
Basal cell hyperplasia or papillae elongation (%)	23 (95.8%)	28 (100%)	0.275
Eosinophilic infiltration (%)	7 (29.2%)	6 (21.4%)	0.521
Petechiae formation (%)	21 (87.5%)	24 (85.7%)	0.851
Hypertrophy of the MM (%)	13 (54.2%)	11 (39.3%)	0.283

Data are presented as mean ± standard deviation or number (percentage). Abbreviations: BMI, body mass index; RDQ, reflux disease questionnaire; HRIM, high resolution impedance manometry; LES, lower esophageal sphincter; IRP 4s, integrated relaxation pressure 4s; MM, muscularis mucosae. $p < 0.05$ indicates statistical significance.

Table S2. Histopathologic comparison between patients with sigmoid and non-sigmoid subtypes of type I achalasia.

	**Sigmoid type**	**Non-sigmoid type**	***p* value**
Number of patients	12	12	
Basal cell hyperplasia or papillae elongation	11 (92.3%)	12 (100%)	0.307
Eosinophilic infiltration	4 (33.3%)	3 (25.0%)	0.653
Petechiae formation	11 (91.7%)	10 (83.3%)	0.537
Hypertrophy of the MM	4 (33.3%)	9 (75.0%)	0.041

Data are presented as number (percentage). Abbreviations: MM, muscularis mucosae. $p < 0.05$ indicates statistical significance.
